# Gender-based time discrepancy in diagnosis of coronary artery disease based on data analytics of electronic medical records

**DOI:** 10.3389/fcvm.2022.969325

**Published:** 2022-11-24

**Authors:** Maryam Panahiazar, Andrew M. Bishara, Yorick Chern, Roohallah Alizadehsani, Sheikh M. Shariful Islam, Dexter Hadley, Rima Arnaout, Ramin E. Beygui

**Affiliations:** ^1^Division of Cardiothoracic Surgery, Department of Surgery, School of Medicine, University of California, San Francisco, San Francisco, CA, United States; ^2^Institute for Computational Health Sciences (ICHS), School of Medicine, University of California, San Francisco, San Francisco, CA, United States; ^3^Department of Anesthesia and Perioperative Care, University of California, San Francisco, San Francisco, CA, United States; ^4^Department of Bioengineering, University of California, Berkeley, Berkeley, CA, United States; ^5^Institute for Intelligent Systems Research and Innovation, Deakin University, Geelong, VIC, Australia; ^6^School of Exercise and Nutrition Sciences, Institute for Physical Activity and Nutrition, Deakin University, Geelong, VIC, Australia; ^7^Department of Clinical Sciences, College of Medicine, University of Central Florida, Orlando, FL, United States; ^8^Division of Cardiology, Department of Medicine, School of Medicine, University of California, San Francisco, San Francisco, CA, United States

**Keywords:** cardiac catheterization, coronary artery bypass grafting (CABG), gender-based discrepancies, Coronary Artery Disease, big data, electronic medical record

## Abstract

**Background:**

Women continue to have worse Coronary Artery Disease (CAD) outcomes than men. The causes of this discrepancy have yet to be fully elucidated. The main objective of this study is to detect gender discrepancies in the diagnosis and treatment of CAD.

**Methods:**

We used data analytics to risk stratify ~32,000 patients with CAD of the total 960,129 patients treated at the UCSF Medical Center over an 8 year period. We implemented a multidimensional data analytics framework to trace patients from admission through treatment to create a path of events. Events are any medications or noninvasive and invasive procedures. The time between events for a similar set of paths was calculated. Then, the average waiting time for each step of the treatment was calculated. Finally, we applied statistical analysis to determine differences in time between diagnosis and treatment steps for men and women.

**Results:**

There is a significant time difference from the first time of admission to diagnostic Cardiac Catheterization between genders (*p-*value = 0.000119), while the time difference from diagnostic Cardiac Catheterization to CABG is not statistically significant.

**Conclusion:**

Women had a significantly longer interval between their first physician encounter indicative of CAD and their first diagnostic cardiac catheterization compared to men. Avoiding this delay in diagnosis may provide more timely treatment and a better outcome for patients at risk. Finally, we conclude by discussing the impact of the study on improving patient care with early detection and managing individual patients at risk of rapid progression of CAD.

## Introduction

Cardiovascular Disease (CVD) encompasses a broad range of conditions and is the leading cause of morbidity and mortality globally and in the United States (1). Despite advances in treatment and survival, CVD is still the leading cause of death among women in the United States (2). Women are less likely to be accurately diagnosed compared to men. A recent study shows sex differences in outcomes after surgery (3). Several non-traditional health occurrences in women predispose them to CVD, including early menopause and menarche, gestational diabetes mellitus, and hypertension. There are discrepancies in gender, ethnicity, race, and age in CVD diagnosis and treatment (4–8). The general guidelines and management of CVD often are similar in most aspects for both genders. Gender-based variations in the pathophysiology, symptomatology, presentation, efficacy of diagnostic tests, and response to pharmacological interventions exist. Our systematic review of gender-based studies of diagnosis and treatment of CVD in the last 20 years shows discrepancies in outcomes of CAD between men and women (7, 9–15). Studies suggest that knowledge and awareness of bias reduce discrimination and therefore our publication will aid in decreasing physician bias. Besides, unique situations germane to women such as pregnancy and hormone therapy make it challenging to diagnose female patients, especially those who are young, with CVD on time (1). However, the causes of these discrepancies have yet to be fully elucidated and require further detailed analysis to design interventions and structures to minimize bias.

The gender differences in CVD outcomes are less well-understood. Regardless of etiology, it is apparent that women have poor outcomes compared to men for Coronary Artery Disease (CAD) in particular (16). A primary reason may be a delay in diagnosis or a different treatment algorithm for patients with chronic CAD as compared to their male counterparts. The main objective and goal of this study are to find discrepancies between women and men using a multidimensional data analytics framework to risk stratify CAD, commonly referred to as Ischemic Heart Disease (IHD), which is a subgroup of the CVD patient population at UCSF. The specific aim of this study is to shed light on the causes of gender discrepancies, and by using path analysis, we uncovered delays and possible gender-based differences in the diagnosis of CAD. The findings of this study will allow us to better identify the systemic causes of discrepancies within CAD treatment and pinpoint the best methods for intervention to reduce them. For example, knowing the delay in time to diagnose Cardiac Catheterization for women suggests a different kind of practice. One of the suggested methods would be for providers to be more detail-oriented and order more aggressive tests for women with symptoms to reduce the delay. This study aims to identify specific gender-based symptoms that are consistent with CAD. For the patients with these symptoms and consistent medical profiles, a timely referral for a noninvasive diagnostic test to assess for early-onset CVD will be made.

The following sections provide an overview of the study design from the definition of the hypothesis to future work. We explain the study cohort followed by data prepossessing, data dictionary, data processing, and data analytics. Next, we show validation and results. The last section discusses the results, study limitations, and next steps. Finally, we conclude the study by discussing its impact on improving patient care with early detection and managing individual patients at risk of rapid progression of CAD.

## Materials and methods

### Study design and overview

We created a cohort selection that allows for simple manipulation and search of the data within the Clinical and Research Data Warehouse (CRDW). This facilitates rapid familiarization and hypothesis testing of the dataset. For example, we hypothesized that there are gender-based discrepancies in the diagnosis and possibly treatment of CAD. With such an extensive patient database and the infrastructure for data abstraction in place at UCSF Bakar Computational Health Sciences Institute, we have been able to describe these discrepancies. We believe that specific studies for individual patients based on medical record profiles with demographic information are more accurate for improving health outcomes in patients with CAD.

**Figure 1:**
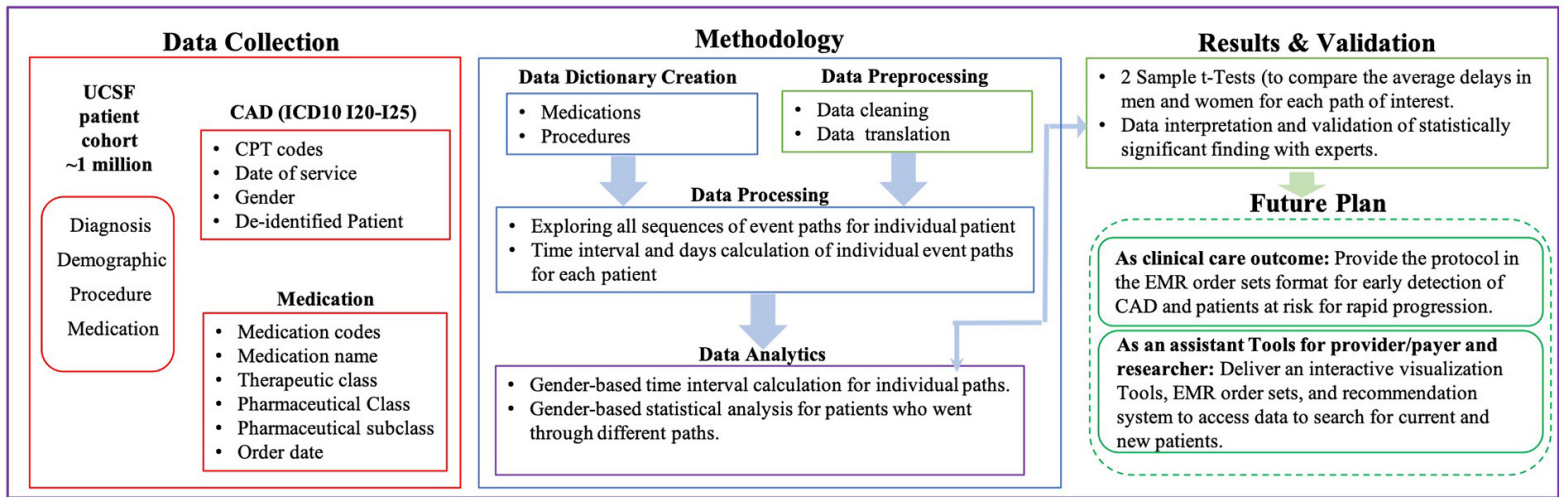
Study overview and architecture. This figure illustrates the major components of the study from data collection to the results and future plan.

This study is designed around the basic workflow considering several steps. It includes hypothesis definition, study cohort, and population, data dictionary creation, data prepossessing, data processing, data analytics, validation and results, and finally future steps. Figure 1 illustrates the major components of the study from hypothesis definition to the future plan. We defined the existence of discrepancies across different genders in the:

- Diagnosis and the time of diagnosis.
- Procedures, including invasive and non-invasive procedures.
- The time interval between diagnosis, medication order, and procedure.

### Study cohort and population

**Figure 2:**
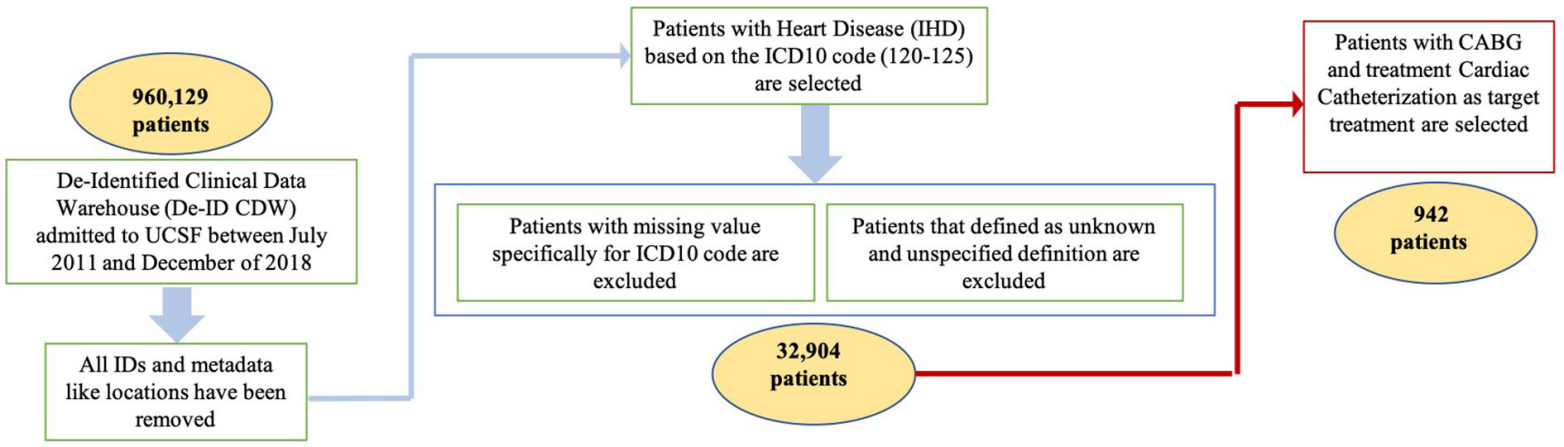
Study cohort selection. This figure illustrates the ste*p-*by-step study cohort selection for including and excluding criteria.

Our data analytics were built using EMR data on 960,129 patients admitted to UCSF between July 2011 and December 2018. This study does not include any human subjects or experimental protocol. All data-based De-Identified Clinical Data Warehouse (De-ID CDW) was authorized to access as “de-identified” by the University of California, San Francisco, and all IDs and metadata (e.g., location) have been removed. All methods were carried out in accordance with relevant guidelines and regulations at UCSF. De-ID CDW is a de-identified database copy of high-value EMR data. Therefore, these data are not subject to HIPAA restrictions on research use, and hence IRB approval or an honest broker intermediary and the need for informed consent was waived by the UCSF Research data team committee. The De-ID CDW system accelerates the research process by permitting UCSF investigators to locate research data and encourage an exploratory approach to hypothesis generation. The De-ID CDW is available to the UCSF research community. The data induced in this research is structural EMR data. The history of patients and clinical notes are excluded from the current study. After authorization to access “de-identified” EMR data for research, in consultation with cardiac, thoracic, and vascular surgeons, cardiologists, and cardiovascular epidemiologists, the following cohort identification criteria were developed as shown in Figure 2:

- Coronary Artery Disease (CAD), commonly referred to as Ischemic Heart Disease (IHD) based on the ICD10 code (120-125). All patients have an initial event at the time of enrollment at UCSF (medication and/or noninvasive procedure). A number of 46,996 patients are selected in this step.
- Patients with missing values (12,600) specifically for the ICD10 code were excluded.
- Patients defined as having an unknown and unspecified definition (1,492) were excluded.

**Figure 3:**
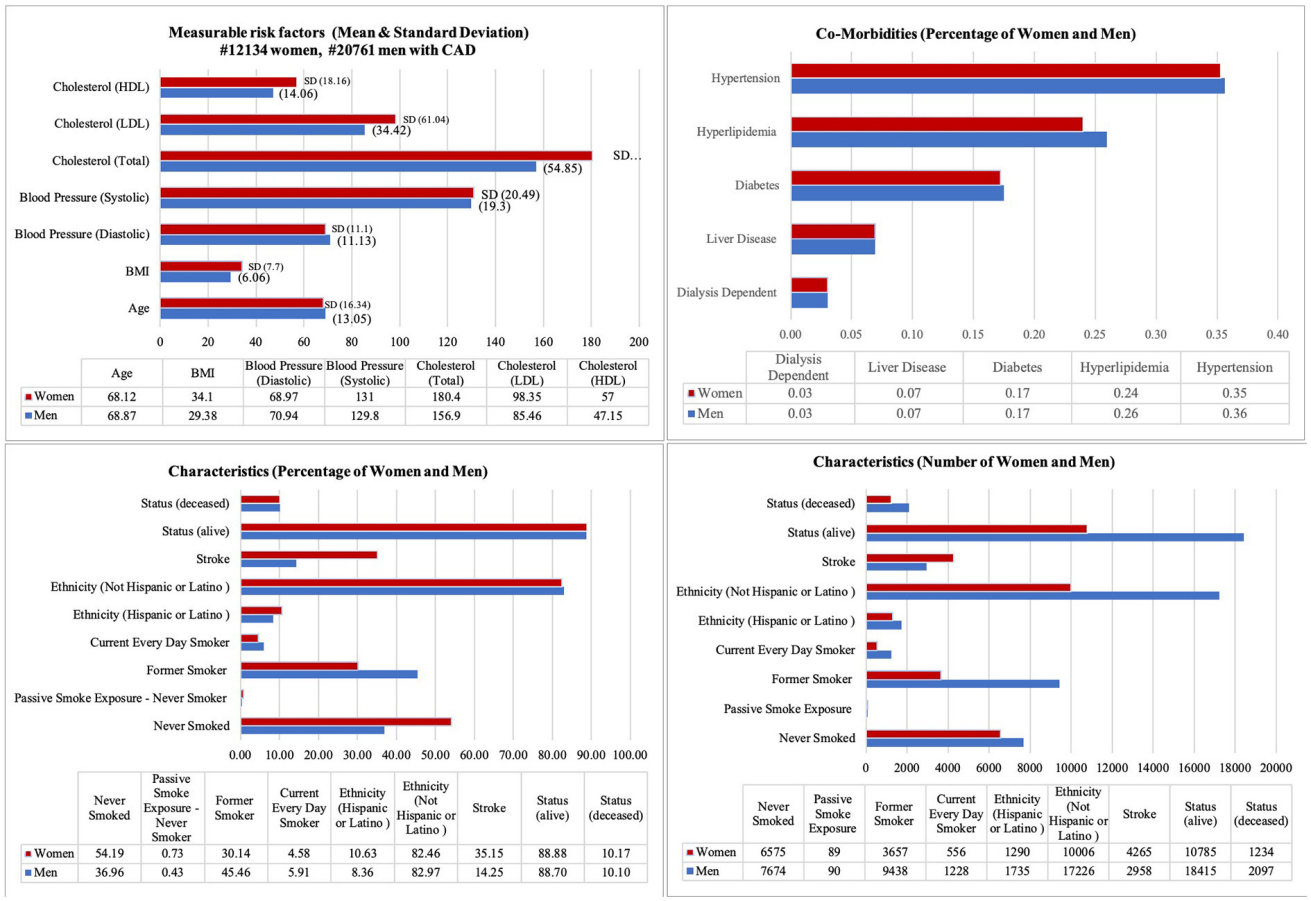
Vital, demographic characteristics and co-morbidities for patients with CAD. It illustrates the mean and standard deviation (SD) for measurable risk factors (e.g., Cholesterol, Blood Pressure, BMI, and Age), Characteristics percentage and number for both gender (e.g., Status, Ethnicity), and Comorbidities [e.g., Hypertension (ICD10 code I10-I15), Dialysis (Z99.2), Diabetes (E08-E13), and Hyperlipidemia (E78.5)].

To be included in this cohort, patients needed to meet the above criteria, leading to a cohort size of 32,904 CAD patients who have been seen by a cardiologist healthcare provider specifically. Since the data was selected from one resource as UCSF, it was no problem with different encounters from different resources to define a longitudinal health record. For multiple initials, if there were several of the same events, we just looked at the first one based on the date of the event. If the patients had two different events on the same day (e.g., Aspirin and EKG test), then we considered the time of the event to select the first event of the path. If there were multiple treatment paths for each patient, then we considered each treatment separately. In each path, we put the earliest event as the first event (e.g., Medication) and then follow-up with the next events (e.g., another Medication, another Procedure) to the end as a sequence of events. Measurable risk factors such as high-density lipoprotein cholesterol (HDL), low-density lipoprotein cholesterol (LDL), cholesterol (TOTAL), systolic blood pressure, diastolic blood pressure, BMI, and age have been considered. Demographic characteristics such as ethnicity have been considered. Smoking conditions including patients never smoked, current everyday smokers, former smokers, and passive smoke exposure are very important characteristics to be considered. It was important to know the gender differences in patients with CAD according to possible comorbidities (e.g., hypertension, liver disease, hyperlipidemia, diabetes, dialysis). Therefore, for each comorbidity, the percentages of women and men are documented. All measurable risk factors, characteristics, and comorbidities are shown in Figure 3. This dataset consisted of de-identified patient ID, demographic information (e.g., gender), and diagnosis based on the ICD10 code as shown in Table 1. The type of angina (unstable, stable, and other types) was based on clinicians' diagnosis and corresponding ICD10 codes as shown in Table 1. The details of ICD codes are described in Supplementary Table S1 (ICD10 details). For procedure code, Current Procedural Terminology (CPT) and the date of procedure services for invasive and non-invasive procedures are used. The medication code, medication name, and date of the orders are used for medications.

**Table 1:** ICD10 I20-I25 for CAD.

**ICD10**	**Definition**	**Subgroups**
I20	Angina pectoris	I20.0, I20.1, I20.8, I20.9
I21	Acute myocardial infarction	I21.0(I21.01,I21.02,I21.09), I21.1(I21.11,I21.19), I21.2(I21.21,I21.29), I21.3, I21.4, I21.9, I21.A(I21.A1,I21.A9)
I22	Subsequent ST elevation and non-ST elevation myocardial infarction	I22.0, I22.1, I22.2, I22.8, I22.9
I23	Certain current complications following ST elevation and non-ST elevation myocardial infarction	I23.0, I23.1, I23.2, I23.3, I23.4, I23.5, I23.6, I23.7, I23.8
I24	Other acute ischemic heart diseases	I24.0, I24.1, I24.8, I24.9
I25	Chronic ischemic heart disease	I25.1, I25.2, I25.3, I25.4, I25.5, I25.6, I25.7, I25.8

Supplementary Table S1 (ICD10 codes) shows all details for ICD10 Codes.

### Data dictionary

A data dictionary for procedures (e.g., CPT codes include diagnostic cardiac catheterization, treatment cardiac catheterization, cardiac CT scan, echo, EKG, myocardial lab, and stress test) is created with the cardio-thoracic surgeons. Refer to Supplementary Table S2 (Procedure Dictionary) for the full list including all codes and names for procedures. To create a dictionary for medication, different medications were classified into the main classes including anticoagulants, antiplatelets, aspirin, beta-blocker, calcium antagonist, cardiac drug, cardiovascular drugs, nitrate, ranolazine, and statin. A dictionary including all codes and names for medications is provided in Supplementary Table S3 (Medication Dictionary). In medication data, 40 medication codes were considered as aspirin including two groups of therapeutic classes defined as analgesics and antiplatelet, which includes groups of medication pharmaceutical classes including analgesic antipyretics, salicylates, analgesics, salicylate, and non-salicylate comb, bulk chemicals, and platelet aggregation inhibitors. These medications are under the medication pharmaceutical sub-classes defined as salicylate analgesics, salicylate analgesics with non-salicylate analgesics combinations, and salicylate analgesics buffered. Our dictionary for complete information about aspirin and classifications is in Supplementary Table S6 (aspirin). These dictionaries are used to create an ontology for cardiothoracic surgical education and clinical data analytics. This ontology can be used for the organization of a variety of concepts, which are used to describe different terms in different resources (17).

### Data processing and statistical analytics

Our approach was based on patient data over time from the date of admission to the date of treatment. Because of the diverse patient cohort at UCSF, each patient is followed from initial interaction/admission/enrollment based on date and time with the UCSF medical system following up any medication order and invasive/noninvasive CAD-related procedures over months and years of treatment. For each patient, the sequence of events was created from the time of initial presentation to the UCSF medical system to the last invasive procedure. Patients are restricted with special targets (CABG and Treatment Cardiac Catheterization) and the exact time of the events. Patients who dropped the follow-up and having incomplete data are excluded to avoid censored data (#1201), as we needed to know the time of each event to make a sequence of events based on the date. To confirm the event has happened and not failed, the diagnosis and procedure code of the event are used rather than the code used for ordering the procedures.

Treatment paths with suspicion of potential cardiovascular disease that combined both procedures and medications are considered. Initially, our experts determined that aspirin is one of the drugs that is frequently ordered early on encountering a patient at risk of cardiovascular disease. Thus, as a first analytic step, we calculated the number of days between the first time aspirin (other medications have been considered too) was prescribed and the first time diagnostic Cardiac Catheterization was recorded, and then from diagnostic Cardiac Catheterization to treatment procedures such as percutaneous coronary intervention (treatment Cardiac Catheterization) and coronary artery bypass graft (CABG). Since aspirin is an over-the-counter medication, there is a high possibility of aspirin not being recorded as a medication order in the EMR. Then, the time between the other first cardiovascular medication order and the first procedure recorded in EMR, not limiting the starting point to aspirin, is calculated. Any type of medication that belongs to the classes of cardiovascular and cardiac drugs, anticoagulants, anti-platelets, aspirin, beta-blockers, and statin are included as the starting point medication for patients suspected to be at risk for cardiovascular disease. If it was a refill period (e.g., for 3 m), it was automatically considered the same medication unless it was prescribed again. The path could be as follows: Aspirin to Aspirin to B-blocker or/and Aspirin to B-blocker.

Our methods are implemented to determine the first suspicion of CAD by providers (primary care and/or cardiologist). Patients with different treatment paths are assigned to separate groups. For example, if the patient had an initial treatment at the time of enrollment at UCSF and started with prescribing aspirin and another patient started the treatment plan with an EKG test, then these two patients are in different groups. Due to this consideration, we found so many treatment plans and paths as is reported in Supplementary Table S5 (All paths). The time between different events (e.g., the time between prescribing aspirin and/or any other medications and ordering the EKG test, EKG test to CABG) is calculated, and the sequence of events for each group of similar patients is created. Similar patients are defined as patients with the same path of events during the treatment. Similar patients have the same starting and ending points and the same sequence of events between these two points. Both medications and procedures are considered over the time of treatment. Then, the sequence of medication orders and procedures over time from the time of admission to the end of treatment are merged. Event time was defined as the date of the first event (e.g., prescribing aspirin, ordering stress test) until the date of the next event (e.g., ordering EKG test) and the next event. All medications and procedures from the dictionary can count as the first event in the patient records. All possible existing events as a pair of event (e.g., aspirin => EKG test, EKG test => diagnostic Cardiac Catheterization, Cardiac Catheterization => CABG) paths for individual patients are considered. Then, the time interval between every two pairs of events and the number of days is calculated. Table 2 shows a few examples of the events. Supplementary Table S4 (Time Intervals) and Supplementary Table S5 (All Paths) show all paths and time intervals for all possible sequences of events.

**Table 2:** Example of the time interval and days between pair of events for a patient.

**Deidentified Patient ID**	**Path of events**	**Time interval**	**Days**
**1(deID PID)	Aspirin ⇒ EKG	[2011-09-05, 2012-01-21]	76
**1(deID PID)	EKG ⇒ diagnostic Cardiac Catheterization	[2012-01-21, 2012-04-11]	80
**1(deID PID)	diagnostic Cardiac Catheterization ⇒ CABG	[2012-04-11, 2012-04-22]	11

Supplementary Table S4 (Time Intervals) shows all paths and time intervals for all possible sequences of events.

The dataset is divided into separate datasets for men and women. For each set, each row with the same “Path” and the compiled days spanned into a list containing different days from different patients are grouped together. Upon the completion of the list of days for each different path, the mean, standard deviation, number of patients, and essentially the length of the day are calculated for both men and women in the dataset. As the very last step, both the men's and women's sets are merged, or concatenated, on the same paths. Then, independent two-sample *t-*Tests are performed for each path to evaluate whether the differences between the average delay days for men and women are statistically significant or not. Usually, the independent samples *t-*Test is used to test whether the population means of two groups are equal or not. The null hypothesis for a two-sample *t-*Test was that the two groups are equal. The two-sample *T-*test assumes that the means of the samples are normally distributed, and it does not assume that the population is normally distributed.

By the Central Limit Theorem, means of samples from a population with finite variance approaches a normal distribution as the sample size increases to infinity regardless of the distribution of the population. The samples are normally distributed as long as the sample size is at least 20. The *T-*test which is based on the mean is not valid for small sample sizes from non-normal distributions, but it is valid for large sample sizes from a non-normal distribution. In our case, our sample size greatly exceeded 20.

The two-sample *T-*test assumes that the means of the samples are normally distributed, and it does not assume that the population is normally distributed.

By the Central Limit Theorem, means of samples from a population with finite variance approaches a normal distribution as the sample size increases to infinity regardless of the distribution of the population. The samples are normally distributed as long as the sample size is at least 20. The *T-*test which is based on the mean is not valid for small sample sizes from non-normal distribution, but it is valid for large sample sizes from a non-normal distribution. In our case, our sample size greatly exceeded 20. The significance level (denoted as α or alpha) of 0.05 is considered. A significance level of 0.05 indicates that a risk of 5% difference exists when there are no real differences. Differences in delay time between groups were assessed with the *p-*value. When the *p-*value is ≤0.05, the null hypothesis that there is no difference can be rejected.

## Results

Table 3 shows a few examples of the results of data analytics. Supplementary Table S5 (All Paths) in supplementary material shows data analytic results for all paths for all patients. Table 4 shows data analytics for all different paths with aspirin as a starting point. Suspecting that the reason for reaching insignificant statistical results is the number of patients who had an order of aspirin as a first encounter recorded was not large enough to reach statistical significance. Table 5 shows the path of all CV medications (as defined in Methods) to diagnostic Cardiac Catheterization, showing that the first medication prescribed to the first diagnostic Cardiac Catheterization ordered is delayed in women compared with men who eventually end up undergoing the treatment Cardiac Catheterization or CABG. With this new starting point, as with all medications, the number of patients (both men and women) increased.

**Table 3:** Example of gender-based time interval calculation for individual pairs of event in the path.

**Path**	**Men**			**Women**			***p-value***
	**Average days**	**SD**	**#** ***n***	**Average** **days**	**SD**	**#** ***n***	
Aspirin ⇒ EKG	160.97	305.17	2,457	178.03	317.29	1,371	0.1060
EKG ⇒ diagnostic Cardiac Catheterization	304.70	471.16	2,010	368.29	496.86	1,033	0.0006
diagnostic Cardiac Catheterization ⇒ CABG	77.06	231.72	237	127.18	329.98	64	0.2570

It includes statistical analysis (average day between events in the path as average days; standard deviation as SD, number of patients as #n, and p-value) for patients who went through the path of interest. The full table is in Supplementary Table S5 (All Paths).

**Table 4:** Example of gender-based data analytics for all pairs in the path with aspirin as a starting point.

**Path**	**Men**			**Women**			***p-value***
	**Average days**	**SD**	**#** ***n***	**Average** **days**	**SD**	**#** ***n***	
aspirin ⇒ anticoagulants	113.56	300.95	2,437	123.84	307.93	1,294	0.3280
aspirin ⇒ antiplatelet	108.51	263.92	1,056	133.02	330.11	434	0.1690
aspirin ⇒ beta-blockers	88.37	237.42	2,492	105.97	258.35	1,268	0.0425
aspirin ⇒ calcium antagonist	231.83	412.29	328	236.84	413.31	230	0.8879
aspirin ⇒ cardiac drugs	180.69	364.89	1,944	193.70	373.32	1,078	0.3552
aspirin ⇒ cardiovascular drugs	106.42	257.81	2,463	138.62	304.98	1,255	0.0013
aspirin ⇒ EKG	160.97	305.17	2,457	178.03	317.29	1,371	0.1060
aspirin ⇒ nitrate	178.89	346.62	1,378	179.45	338.49	757	0.9713
aspirin ⇒ ranolazine	238.98	396.04	70	387.89	490.56	37	0.1164
aspirin ⇒ statin	99.68	247.01	2,549	115.03	264.83	1,276	0.0839
aspirin ⇒ CABG	276.96	418.96	94	358.07	508.62	28	0.4462
aspirin ⇒ diagnostic Cardiac Catheterization	300.71	442.23	509	347.77	472.48	252	0.1873
aspirin ⇒ treatment Cardiac Catheterization	267.83	427.68	212	347.72	516.78	69	0.2482

It includes two-sample t-Tests to compare the average delay in men and women for each path of interest. A complete table is in Supplementary Table S6 (Aspirin).

**Table 5:** Example of gender-based data analytics for all cardiovascular related medications as starting point of the treatment plan.

**Path**	**Men**		**Women**		***p-value***
	**Average** **interval days**	**# Patient**	**Average** **interval days**	**#Patient**	
all Cardiovascular Medications ⇒ CABG	414.25	157	436.28	47	0.8096
all Cardiovascular Medications ⇒ diagnostic Cardiac Catheterization	395.67	884	457.76	444	0.0482
all Cardiovascular Medications ⇒ treatment Cardiac Catheterization	419.69	298	539.53	95	0.0920

The medications belong to the classes of cardiovascular drugs, cardiac drugs, anticoagulants, anti-platelets, aspirin, beta-blockers, and statin. The complete table is published in Supplementary Table S5 (All Paths).

The results show the path between the very first event (medication, noninvasive procedure) and the next event (medication, noninvasive, and invasive procedure), medication (e.g., statin), and procedures (e.g., treatment Cardiac Catheterization). All medications and procedures are selected from our defined dictionaries with experts, which are explained earlier. Dictionaries are available in Supplementary Table S3 (Medication Dictionary) and Supplementary Table S2 (Procedure Dictionary). Of patients who ultimately underwent a therapeutic intervention—CABG or Treatment Cardiac Catheterization—there was a greater delay in time to diagnose Cardiac Catheterization for women. Performing a two-sample *t-*Test on time to diagnostic Catheterization between men and women showed statistical significance when compared against a threshold of 0.05 (however, even when compared to the Bonferroni corrected threshold of 0.05/181 = 0.00028 where 181 is the number of paths, it shows that the delays between the path of the first event to diagnostic cardiac catheterization are still significant with a *p-*value of 0.00019), meaning that there is indeed a delay in women who eventually undergo therapeutic procedures for CAD to get diagnostic Cardiac Catheterization. In summary, there is a significant time difference from the first event to diagnostic Cardiac Catheterization between genders (*p-*value = 0.000119), while the *p-*value for diagnostic Cardiac Catheterization to CABG is not statistically significant. This result is a validation of the hypothesis that there are discrepancies within cardiovascular diagnosis in women and men. It shows that there is a delay to diagnostic Cardiac Catheterization in women who eventually undergo treatment for CAD (Therapeutic Cardiac Catheterization or CABG). With clear results that show the discrepancies in diagnostic procedures in women vs. men, in the next section, the possible implications of this study on patient care will be discussed.

## Discussion

This study shows the use of data analytics to reveal gender-based discrepancies in the diagnosis and treatment of CAD. One of the novelties of this study is tracing a multidimensional aspect of patients' diagnosis and treatment over time. All related events (e.g., prescribing medication, test, procedure) are reviewed over the time of diagnosis and treatment. Recognizing a clear delay in diagnosis (i.e., time to diagnostic catheterization in women) will make a change in practice and will result in improved outcomes for women with CAD with early detection. The time interval between different events (e.g., the time between prescribing medication and ordering the cardiac stress test) is measured. Then, the sequence of events for each patient and group of similar patients are extracted. Our results, based on the analysis of a subset of patients with CAD conditions, support the hypothesis of existing discrepancies in the diagnosis of CAD based on patient demographic characteristics, such as gender.

The results show that when women with the eventual diagnosis of severe CAD are started on aspirin, it takes them longer to start beta-blockers, a known drug to reduce cardiovascular risk, compared to men. Women who have undergone CABG, on average, have waited for 358 to get the “Gold Standard” diagnostic Cardiac Catheterization followed by an extra 127 days to undergo CABG for severe CAD. Men who have undergone CABG on average waited for 291 to get the “Gold Standard” diagnostic Cardiac Catheterization followed by extra 77 days to undergo CABG for severe CAD. From a starting point of any first event (e.g., aspirin order, cardiac stress test order), on average, it takes over 2 months for women to undergo CABG compared to men. In patients with left main and multivessel CAD or unstable angina, the risk of a CAD event is high. For example, if 50% are at risk of some event in 6 months (ACS, STEMI, NSTEMI, or sudden cardiac death), then it can be extrapolated that a delay of 2 months would result in a 17% increased risk for women compared to men. Our goal was to simplify hypothesis testing as much as possible for healthcare providers and researchers. This study shows that the kind of data analytics, which has been used in this study, is sufficient to find the discrepancies within the cardiovascular diagnosis. While our work focused on the UCSF data, we anticipate that our approach can be applied to other databases of patient data with similar levels of success (e.g., UC System-wide data). Based on our analysis, the difference in the interval from the first event to diagnostic Cardiac Catheterization is the intervention with a significant *p-*value of 0.000119, while the *p-*value for diagnostic Cardiac Catheterization to CABG and diagnostic Cardiac Catheterization to treatment Cardiac Catheterization is not statistically significant.

In summary, our research has important implications for initiatives aimed at improving the use of EMR to find the possible reasons for different outcomes in women vs. men or based on differences in other patient characteristics. Several efforts are devoted to finding the different outcomes and risk factors in different genders, but the reasons for the differences are not yet fully identified. The focus of this study was to find the possible reasons or one of the reasons for discrepancies. In ongoing research, we are looking at outcomes in different genders specifically survival analysis, readmission for the same issue, stroke, and changes in Ejection Fraction (EF) after invasive procedures in different genders.

For some patients, the code for diagnostic Catheterization was not entered. As a result of this limitation, it was a decrease in the number of patients with the path from diagnostic Cardiac Catheterization to CABG. Although the number of patients who had the starting point of CAD on admission to UCSF before was ~32,000 after cleaning, a small subset of them went to treatment therapeutic Catheterization and CABG from diagnostic Cardiac Catheterization. This specific target dropped the number of patients to 942, as shown in Figure 2. We plan to use UC system data to cover more patients with these two targets to overcome this limitation. The number of targets will be extended to more invasive procedures. Also, the feeling and experience of each doctor could be different and a patient with suspected CAD may not necessarily have started their treatment with aspirin or any other cardiovascular medication. Moreover, given the high prevalence of non-invasive false-positive tests and atypical symptoms in women, many physicians choose not to perform cardiac catheterization initially, but to perform coronary CT angiography. This may explain another possible reason for the delay in the treatment of CAD in women vs. men. When features and predictive variables are different in men and women, decision-making based on the unified platforms and guidelines for the diagnosis and treatment of the patients appears to lead to poor outcomes in women compared to men. Therefore, studies on CAD based on individual characteristics (e.g., demographics) will have a big impact on the diagnosis and treatment of CAD. We plan to find a patient profile that describes rapidly progressive CAD and flag these patients for frequent and regular cardiovascular assessment. Interactive visualization tools will be developed for providers, payers, and researchers to assist the personalized treatment plans for individual patients with specific characteristics based on new guidelines and suggestions as EMR order sets.

Our future goal is to translate the multi-dimensional big data including EMR that is generated at the University of California System to directly improve and assist clinical care decision-making that ultimately would improve outcomes for patients and reduce cost. We will look at other demographic information, such as sex diversity (gay, lesbian, etc.). Moreover, this study lays the foundation to develop novel translational interventions through powerful big data-driven analytics that leverage the wide availability of UC System patient data. As an implementation of clinical care, this study's goal is to improve precision diagnosis and ultimately, management of CVD for both early detection and identification of patients at risk for rapid progression of the disease. As a clinical care outcome, a protocol in the EMR order sets format will be provided for early detection of severe CAD in patients at risk for rapid progression. As an example, for a woman with a history of hormone therapy, pregnancy with hypertension at an early age, family history, and increased BMI, we suggest expediting the more sensitive testing (stratified and varied order sets depending on that patient's risk profile) instead of long-term therapy with medications (e.g., aspirin, statins, beta-blockers) and diagnose the CAD expeditiously. As an assistant tool for providers, payers, and researchers, we plan to deliver interactive visualization Tools, updated EMR order sets, and recommendation systems to access data to search and use the guidelines for the treatment of individual patients with specific characteristics. The outcome of this research lays the foundation to develop novel translational interventions through powerful big data-driven analytics that leverage the wide availability of UC System patient data.

## Conclusion

Although the overall guidelines and management of CAD are similar for both genders, gender-based variations in the pathophysiology, symptomatology, presentation, efficacy of diagnostic tests, and response to pharmacological interventions do exist. There are discrepancies in the delivery of healthcare in general across different genders. Women with severe CAD requiring revascularization have a significantly longer interval between their first physician encounter indicative of cardiovascular disease to their first diagnostic Cardiac Catheterization compared to men. These differences in healthcare delivery, methodology, diagnosis procedure, and the time interval between diagnosis procedure and therapeutics may have a significant impact on a patient's health and outcome with early detection of the problem. Personalized treatment for individuals based on specific EMR profiles and demographic characteristics may significantly reduce unnecessary treatments and costs, and potentially, morbidity and mortality of downstream procedures associated with wrong or late diagnosis. Moreover, improved precision may change the debate surrounding the standard guidelines based on gender and other individual characteristics of the patients. Developing updated gender-based guidelines will help providers for both early detection and managing individual patients at risk of rapid progression of CAD and generally in CVD. This new guideline will be an innovation in clinical care. Avoiding delays in diagnosis may provide more timely treatment and a better outcome for patients who are at risk.

## Data availability statement

The original contributions presented in the study are included in the article/Supplementary material, further inquiries can be directed to the corresponding author/s.

## Ethics statement

Ethical review and approval was not required for the study on human participants in accordance with the local legislation and institutional requirements. Written informed consent from the patients was not required to participate in this study in accordance with the national legislation and the institutional requirements.

## Author contributions

MP, RB, DH, and AB designed and discussed the research studies and analyzed the results. MP, RB, and AB defined and selected data for the study cohort. MP, RB, AB, YC, and DH implemented a big data analytics platform. MP, RB, YC, and AB wrote the manuscript. RA and MP provided a systematic review of related works. MP, RB, RA, and SI edited and revised the manuscript. All authors contributed to the article and approved the submitted version.

## Funding

This research was supported by the UCSF Department of Surgery Research Fund and the National Library of Medicine of the NIH (under Award Number U01LM012675 and T32 NIH funding 5T32GM008440).

## Conflict of interest

The authors declare that the research was conducted in the absence of any commercial or financial relationships that could be construed as a potential conflict of interest.

## Publisher's note

All claims expressed in this article are solely those of the authors and do not necessarily represent those of their affiliated organizations, or those of the publisher, the editors and the reviewers. Any product that may be evaluated in this article, or claim that may be made by its manufacturer, is not guaranteed or endorsed by the publisher.

## Author disclaimer

The content is solely the responsibility of the authors and does not necessarily represent the official views of the NIH.
