# Supplementary material for: Gender-based time discrepancy in diagnosis of coronary artery disease based on data analytics of electronic medical records
Source: Front Cardiovasc Med. 2022 Nov 24;9:969325. doi: 10.3389/fcvm.2022.969325 (PMC9729739; doi:10.3389/fcvm.2022.969325)
Supplement: Supplementary file 1 [file Table_1.DOCX]

- [I20-I25](https://www.icd10data.com/ICD10CM/Codes/I00-I99/I20-I25) Ischemic heart diseases

Codes

- [I20](https://www.icd10data.com/ICD10CM/Codes/I00-I99/I20-I25/I20-)
- Angina pectoris
- [I21](https://www.icd10data.com/ICD10CM/Codes/I00-I99/I20-I25/I21-)
- Acute myocardial infarction
- [I22](https://www.icd10data.com/ICD10CM/Codes/I00-I99/I20-I25/I22-)
- Subsequent ST elevation (STEMI) and non-ST elevation (NSTEMI) myocardial infarction
- [I23](https://www.icd10data.com/ICD10CM/Codes/I00-I99/I20-I25/I23-)
- Certain current complications following ST elevation (STEMI) and non-ST elevation (NSTEMI) myocardial infarction (within the 28 day period)
- [I24](https://www.icd10data.com/ICD10CM/Codes/I00-I99/I20-I25/I24-)
- Other acute ischemic heart diseases
- [I25](https://www.icd10data.com/ICD10CM/Codes/I00-I99/I20-I25/I25-)
- Chronic ischemic heart disease
- [I20](https://www.icd10data.com/ICD10CM/Codes/I00-I99/I20-I25/I20-/I20) Angina pectoris
  - [I20.0](https://www.icd10data.com/ICD10CM/Codes/I00-I99/I20-I25/I20-/I20.0) Unstable angina
  - [I20.1](https://www.icd10data.com/ICD10CM/Codes/I00-I99/I20-I25/I20-/I20.1) Angina pectoris with documented spasm
  - [I20.8](https://www.icd10data.com/ICD10CM/Codes/I00-I99/I20-I25/I20-/I20.8) Other forms of angina pectoris
  - [I20.9](https://www.icd10data.com/ICD10CM/Codes/I00-I99/I20-I25/I20-/I20.9) Angina pectoris, unspecified

Codes

- [I21](https://www.icd10data.com/ICD10CM/Codes/I00-I99/I20-I25/I21-/I21) Acute myocardial infarction
  - [I21.0](https://www.icd10data.com/ICD10CM/Codes/I00-I99/I20-I25/I21-/I21.0) ST elevation (STEMI) myocardial infarction of anterior wall
    - [I21.01](https://www.icd10data.com/ICD10CM/Codes/I00-I99/I20-I25/I21-/I21.01) ST elevation (STEMI) myocardial infarction involving left main coronary artery
    - [I21.02](https://www.icd10data.com/ICD10CM/Codes/I00-I99/I20-I25/I21-/I21.02) ST elevation (STEMI) myocardial infarction involving left anterior descending coronary artery
    - [I21.09](https://www.icd10data.com/ICD10CM/Codes/I00-I99/I20-I25/I21-/I21.09) ST elevation (STEMI) myocardial infarction involving other coronary artery of anterior wall
  - [I21.1](https://www.icd10data.com/ICD10CM/Codes/I00-I99/I20-I25/I21-/I21.1) ST elevation (STEMI) myocardial infarction of inferior wall
    - [I21.11](https://www.icd10data.com/ICD10CM/Codes/I00-I99/I20-I25/I21-/I21.11) ST elevation (STEMI) myocardial infarction involving right coronary artery
    - [I21.19](https://www.icd10data.com/ICD10CM/Codes/I00-I99/I20-I25/I21-/I21.19) ST elevation (STEMI) myocardial infarction involving other coronary artery of inferior wall
  - [I21.2](https://www.icd10data.com/ICD10CM/Codes/I00-I99/I20-I25/I21-/I21.2) ST elevation (STEMI) myocardial infarction of other sites
    - [I21.21](https://www.icd10data.com/ICD10CM/Codes/I00-I99/I20-I25/I21-/I21.21) ST elevation (STEMI) myocardial infarction involving left circumflex coronary artery
    - [I21.29](https://www.icd10data.com/ICD10CM/Codes/I00-I99/I20-I25/I21-/I21.29) ST elevation (STEMI) myocardial infarction involving other sites
  - [I21.3](https://www.icd10data.com/ICD10CM/Codes/I00-I99/I20-I25/I21-/I21.3) ST elevation (STEMI) myocardial infarction of unspecified site
  - [I21.4](https://www.icd10data.com/ICD10CM/Codes/I00-I99/I20-I25/I21-/I21.4) Non-ST elevation (NSTEMI) myocardial infarction
  - [I21.9](https://www.icd10data.com/ICD10CM/Codes/I00-I99/I20-I25/I21-/I21.9) Acute myocardial infarction, unspecified
  - [I21.A](https://www.icd10data.com/ICD10CM/Codes/I00-I99/I20-I25/I21-/I21.A) Other type of myocardial infarction
    - [I21.A1](https://www.icd10data.com/ICD10CM/Codes/I00-I99/I20-I25/I21-/I21.A1) Myocardial infarction type 2
    - [I21.A9](https://www.icd10data.com/ICD10CM/Codes/I00-I99/I20-I25/I21-/I21.A9) Other myocardial infarction type
- [I22](https://www.icd10data.com/ICD10CM/Codes/I00-I99/I20-I25/I22-/I22) Subsequent ST elevation (STEMI) and non-ST elevation (NSTEMI) myocardial infarction
  - [I22.0](https://www.icd10data.com/ICD10CM/Codes/I00-I99/I20-I25/I22-/I22.0) Subsequent ST elevation (STEMI) myocardial infarction of anterior wall
  - [I22.1](https://www.icd10data.com/ICD10CM/Codes/I00-I99/I20-I25/I22-/I22.1) Subsequent ST elevation (STEMI) myocardial infarction of inferior wall
  - [I22.2](https://www.icd10data.com/ICD10CM/Codes/I00-I99/I20-I25/I22-/I22.2) Subsequent non-ST elevation (NSTEMI) myocardial infarction
  - [I22.8](https://www.icd10data.com/ICD10CM/Codes/I00-I99/I20-I25/I22-/I22.8) Subsequent ST elevation (STEMI) myocardial infarction of other sites
  - [I22.9](https://www.icd10data.com/ICD10CM/Codes/I00-I99/I20-I25/I22-/I22.9) Subsequent ST elevation (STEMI) myocardial infarction of unspecified site

Codes

- [I23](https://www.icd10data.com/ICD10CM/Codes/I00-I99/I20-I25/I23-/I23) Certain current complications following ST elevation (STEMI) and non-ST elevation (NSTEMI) myocardial infarction (within the 28 day period)
  - [I23.0](https://www.icd10data.com/ICD10CM/Codes/I00-I99/I20-I25/I23-/I23.0) Hemopericardium as current complication following acute myocardial infarction
  - [I23.1](https://www.icd10data.com/ICD10CM/Codes/I00-I99/I20-I25/I23-/I23.1) Atrial septal defect as current complication following acute myocardial infarction
  - [I23.2](https://www.icd10data.com/ICD10CM/Codes/I00-I99/I20-I25/I23-/I23.2) Ventricular septal defect as current complication following acute myocardial infarction
  - [I23.3](https://www.icd10data.com/ICD10CM/Codes/I00-I99/I20-I25/I23-/I23.3) Rupture of cardiac wall without hemopericardium as current complication following acute myocardial infarction
  - [I23.4](https://www.icd10data.com/ICD10CM/Codes/I00-I99/I20-I25/I23-/I23.4) Rupture of chordae tendineae as current complication following acute myocardial infarction
  - [I23.5](https://www.icd10data.com/ICD10CM/Codes/I00-I99/I20-I25/I23-/I23.5) Rupture of papillary muscle as current complication following acute myocardial infarction
  - [I23.6](https://www.icd10data.com/ICD10CM/Codes/I00-I99/I20-I25/I23-/I23.6) Thrombosis of atrium, auricular appendage, and ventricle as current complications following acute myocardial infarction
  - [I23.7](https://www.icd10data.com/ICD10CM/Codes/I00-I99/I20-I25/I23-/I23.7) Postinfarction angina
  - [I23.8](https://www.icd10data.com/ICD10CM/Codes/I00-I99/I20-I25/I23-/I23.8) Other current complications following acute myocardial infarction

Codes

- [I24](https://www.icd10data.com/ICD10CM/Codes/I00-I99/I20-I25/I24-/I24) Other acute ischemic heart diseases
  - [I24.0](https://www.icd10data.com/ICD10CM/Codes/I00-I99/I20-I25/I24-/I24.0) Acute coronary thrombosis not resulting in myocardial infarction
  - [I24.1](https://www.icd10data.com/ICD10CM/Codes/I00-I99/I20-I25/I24-/I24.1) Dressler's syndrome
  - [I24.8](https://www.icd10data.com/ICD10CM/Codes/I00-I99/I20-I25/I24-/I24.8) Other forms of acute ischemic heart disease
  - [I24.9](https://www.icd10data.com/ICD10CM/Codes/I00-I99/I20-I25/I24-/I24.9) Acute ischemic heart disease, unspecified

Codes

- [I25](https://www.icd10data.com/ICD10CM/Codes/I00-I99/I20-I25/I25-/I25) Chronic ischemic heart disease
  - [I25.1](https://www.icd10data.com/ICD10CM/Codes/I00-I99/I20-I25/I25-/I25.1) Atherosclerotic heart disease of native coronary artery
    - [I25.10](https://www.icd10data.com/ICD10CM/Codes/I00-I99/I20-I25/I25-/I25.10) …… without angina pectoris
    - [I25.11](https://www.icd10data.com/ICD10CM/Codes/I00-I99/I20-I25/I25-/I25.11) Atherosclerotic heart disease of native coronary artery with angina pectoris
      - [I25.110](https://www.icd10data.com/ICD10CM/Codes/I00-I99/I20-I25/I25-/I25.110) Atherosclerotic heart disease of native coronary artery with unstable angina pectoris
      - [I25.111](https://www.icd10data.com/ICD10CM/Codes/I00-I99/I20-I25/I25-/I25.111) …… with documented spasm
      - [I25.118](https://www.icd10data.com/ICD10CM/Codes/I00-I99/I20-I25/I25-/I25.118) Atherosclerotic heart disease of native coronary artery with other forms of angina pectoris
      - [I25.119](https://www.icd10data.com/ICD10CM/Codes/I00-I99/I20-I25/I25-/I25.119) Atherosclerotic heart disease of native coronary artery with unspecified angina pectoris
  - [I25.2](https://www.icd10data.com/ICD10CM/Codes/I00-I99/I20-I25/I25-/I25.2) Old myocardial infarction
  - [I25.3](https://www.icd10data.com/ICD10CM/Codes/I00-I99/I20-I25/I25-/I25.3) Aneurysm of heart
  - [I25.4](https://www.icd10data.com/ICD10CM/Codes/I00-I99/I20-I25/I25-/I25.4) Coronary artery aneurysm and dissection
    - [I25.41](https://www.icd10data.com/ICD10CM/Codes/I00-I99/I20-I25/I25-/I25.41) Coronary artery aneurysm
    - [I25.42](https://www.icd10data.com/ICD10CM/Codes/I00-I99/I20-I25/I25-/I25.42) Coronary artery dissection
  - [I25.5](https://www.icd10data.com/ICD10CM/Codes/I00-I99/I20-I25/I25-/I25.5) Ischemic cardiomyopathy
  - [I25.6](https://www.icd10data.com/ICD10CM/Codes/I00-I99/I20-I25/I25-/I25.6) Silent myocardial ischemia
  - [I25.7](https://www.icd10data.com/ICD10CM/Codes/I00-I99/I20-I25/I25-/I25.7) Atherosclerosis of coronary artery bypass graft(s) and coronary artery of transplanted heart with angina pectoris
    - [I25.70](https://www.icd10data.com/ICD10CM/Codes/I00-I99/I20-I25/I25-/I25.70) Atherosclerosis of coronary artery bypass graft(s), unspecified, with angina pectoris
      - [I25.700](https://www.icd10data.com/ICD10CM/Codes/I00-I99/I20-I25/I25-/I25.700) Atherosclerosis of coronary artery bypass graft(s), unspecified, with unstable angina pectoris
      - [I25.701](https://www.icd10data.com/ICD10CM/Codes/I00-I99/I20-I25/I25-/I25.701) …… with documented spasm
      - [I25.708](https://www.icd10data.com/ICD10CM/Codes/I00-I99/I20-I25/I25-/I25.708) Atherosclerosis of coronary artery bypass graft(s), unspecified, with other forms of angina pectoris
      - [I25.709](https://www.icd10data.com/ICD10CM/Codes/I00-I99/I20-I25/I25-/I25.709) Atherosclerosis of coronary artery bypass graft(s), unspecified, with unspecified angina pectoris
    - [I25.71](https://www.icd10data.com/ICD10CM/Codes/I00-I99/I20-I25/I25-/I25.71) Atherosclerosis of autologous vein coronary artery bypass graft(s) with angina pectoris
      - [I25.710](https://www.icd10data.com/ICD10CM/Codes/I00-I99/I20-I25/I25-/I25.710) Atherosclerosis of autologous vein coronary artery bypass graft(s) with unstable angina pectoris
      - [I25.711](https://www.icd10data.com/ICD10CM/Codes/I00-I99/I20-I25/I25-/I25.711) …… with documented spasm
      - [I25.718](https://www.icd10data.com/ICD10CM/Codes/I00-I99/I20-I25/I25-/I25.718) Atherosclerosis of autologous vein coronary artery bypass graft(s) with other forms of angina pectoris
      - [I25.719](https://www.icd10data.com/ICD10CM/Codes/I00-I99/I20-I25/I25-/I25.719) Atherosclerosis of autologous vein coronary artery bypass graft(s) with unspecified angina pectoris
    - [I25.72](https://www.icd10data.com/ICD10CM/Codes/I00-I99/I20-I25/I25-/I25.72) Atherosclerosis of autologous artery coronary artery bypass graft(s) with angina pectoris
      - [I25.720](https://www.icd10data.com/ICD10CM/Codes/I00-I99/I20-I25/I25-/I25.720) Atherosclerosis of autologous artery coronary artery bypass graft(s) with unstable angina pectoris
      - [I25.721](https://www.icd10data.com/ICD10CM/Codes/I00-I99/I20-I25/I25-/I25.721) …… with documented spasm
      - [I25.728](https://www.icd10data.com/ICD10CM/Codes/I00-I99/I20-I25/I25-/I25.728) Atherosclerosis of autologous artery coronary artery bypass graft(s) with other forms of angina pectoris
      - [I25.729](https://www.icd10data.com/ICD10CM/Codes/I00-I99/I20-I25/I25-/I25.729) Atherosclerosis of autologous artery coronary artery bypass graft(s) with unspecified angina pectoris
    - [I25.73](https://www.icd10data.com/ICD10CM/Codes/I00-I99/I20-I25/I25-/I25.73) Atherosclerosis of nonautologous biological coronary artery bypass graft(s) with angina pectoris
      - [I25.730](https://www.icd10data.com/ICD10CM/Codes/I00-I99/I20-I25/I25-/I25.730) Atherosclerosis of nonautologous biological coronary artery bypass graft(s) with unstable angina pectoris
      - [I25.731](https://www.icd10data.com/ICD10CM/Codes/I00-I99/I20-I25/I25-/I25.731) …… with documented spasm
      - [I25.738](https://www.icd10data.com/ICD10CM/Codes/I00-I99/I20-I25/I25-/I25.738) Atherosclerosis of nonautologous biological coronary artery bypass graft(s) with other forms of angina pectoris
      - [I25.739](https://www.icd10data.com/ICD10CM/Codes/I00-I99/I20-I25/I25-/I25.739) Atherosclerosis of nonautologous biological coronary artery bypass graft(s) with unspecified angina pectoris
    - [I25.75](https://www.icd10data.com/ICD10CM/Codes/I00-I99/I20-I25/I25-/I25.75) Atherosclerosis of native coronary artery of transplanted heart with angina pectoris
      - [I25.750](https://www.icd10data.com/ICD10CM/Codes/I00-I99/I20-I25/I25-/I25.750) Atherosclerosis of native coronary artery of transplanted heart with unstable angina
      - [I25.751](https://www.icd10data.com/ICD10CM/Codes/I00-I99/I20-I25/I25-/I25.751) …… with documented spasm
      - [I25.758](https://www.icd10data.com/ICD10CM/Codes/I00-I99/I20-I25/I25-/I25.758) Atherosclerosis of native coronary artery of transplanted heart with other forms of angina pectoris
      - [I25.759](https://www.icd10data.com/ICD10CM/Codes/I00-I99/I20-I25/I25-/I25.759) Atherosclerosis of native coronary artery of transplanted heart with unspecified angina pectoris
    - [I25.76](https://www.icd10data.com/ICD10CM/Codes/I00-I99/I20-I25/I25-/I25.76) Atherosclerosis of bypass graft of coronary artery of transplanted heart with angina pectoris
      - [I25.760](https://www.icd10data.com/ICD10CM/Codes/I00-I99/I20-I25/I25-/I25.760) Atherosclerosis of bypass graft of coronary artery of transplanted heart with unstable angina
      - [I25.761](https://www.icd10data.com/ICD10CM/Codes/I00-I99/I20-I25/I25-/I25.761) …… with documented spasm
      - [I25.768](https://www.icd10data.com/ICD10CM/Codes/I00-I99/I20-I25/I25-/I25.768) Atherosclerosis of bypass graft of coronary artery of transplanted heart with other forms of angina pectoris
      - [I25.769](https://www.icd10data.com/ICD10CM/Codes/I00-I99/I20-I25/I25-/I25.769) Atherosclerosis of bypass graft of coronary artery of transplanted heart with unspecified angina pectoris
    - [I25.79](https://www.icd10data.com/ICD10CM/Codes/I00-I99/I20-I25/I25-/I25.79) Atherosclerosis of other coronary artery bypass graft(s) with angina pectoris
      - [I25.790](https://www.icd10data.com/ICD10CM/Codes/I00-I99/I20-I25/I25-/I25.790) Atherosclerosis of other coronary artery bypass graft(s) with unstable angina pectoris
      - [I25.791](https://www.icd10data.com/ICD10CM/Codes/I00-I99/I20-I25/I25-/I25.791) …… with documented spasm
      - [I25.798](https://www.icd10data.com/ICD10CM/Codes/I00-I99/I20-I25/I25-/I25.798) Atherosclerosis of other coronary artery bypass graft(s) with other forms of angina pectoris
      - [I25.799](https://www.icd10data.com/ICD10CM/Codes/I00-I99/I20-I25/I25-/I25.799) Atherosclerosis of other coronary artery bypass graft(s) with unspecified angina pectoris
  - [I25.8](https://www.icd10data.com/ICD10CM/Codes/I00-I99/I20-I25/I25-/I25.8) Other forms of chronic ischemic heart disease
    - [I25.81](https://www.icd10data.com/ICD10CM/Codes/I00-I99/I20-I25/I25-/I25.81) Atherosclerosis of other coronary vessels without angina pectoris
      - [I25.810](https://www.icd10data.com/ICD10CM/Codes/I00-I99/I20-I25/I25-/I25.810) Atherosclerosis of coronary artery bypass graft(s) without angina pectoris
      - [I25.811](https://www.icd10data.com/ICD10CM/Codes/I00-I99/I20-I25/I25-/I25.811) Atherosclerosis of native coronary artery of transplanted heart without angina pectoris
      - [I25.812](https://www.icd10data.com/ICD10CM/Codes/I00-I99/I20-I25/I25-/I25.812) Atherosclerosis of bypass graft of coronary artery of transplanted heart without angina pectoris
    - [I25.82](https://www.icd10data.com/ICD10CM/Codes/I00-I99/I20-I25/I25-/I25.82) Chronic total occlusion of coronary artery
    - [I25.83](https://www.icd10data.com/ICD10CM/Codes/I00-I99/I20-I25/I25-/I25.83) Coronary atherosclerosis due to lipid rich plaque
    - [I25.84](https://www.icd10data.com/ICD10CM/Codes/I00-I99/I20-I25/I25-/I25.84) Coronary atherosclerosis due to calcified coronary lesion
    - [I25.89](https://www.icd10data.com/ICD10CM/Codes/I00-I99/I20-I25/I25-/I25.89) Other forms of chronic ischemic heart disease

[I25.9](https://www.icd10data.com/ICD10CM/Codes/I00-I99/I20-I25/I25-/I25.9) Chronic ischemic heart disease, unspecified
